# Oxford COVID-19 Vaccine Hesitancy in School Principals: Impacts of Gender, Well-Being, and Coronavirus-Related Health Literacy

**DOI:** 10.3390/vaccines9090985

**Published:** 2021-09-03

**Authors:** Tuyen Van Duong, Cheng-Yu Lin, Sheng-Chih Chen, Yung-Kai Huang, Orkan Okan, Kevin Dadaczynski, Chih-Feng Lai

**Affiliations:** 1 School of Nutrition and Health Sciences, Taipei Medical University, Taipei 110-31, Taiwan; tvduong@tmu.edu.tw; 2 Department of Radio, Television & Film, Shih Hsin University, Taipei 116-42, Taiwan; cyou.lin@msa.hinet.net; 3 Master’s Program of Digital Content and Technologies, College of Communication, National Chengchi University, Taipei 116-05, Taiwan; scchen@nccu.edu.tw; 4 Department of Oral Hygiene, College of Dental Medicine, Kaohsiung Medical University, Kaohsiung 807-08, Taiwan; ykhuang@kmu.edu.tw; 5 Interdisciplinary Centre for Health Literacy Research, Faculty of Educational Science, Bielefeld University, 33615 Bielefeld, Germany; orkan.okan@uni-bielefeld.de; 6 Public Health Centre Fulda, Fulda University of Applied Sciences, 36037 Fulda, Germany; kevin.dadaczynski@pg.hs-fulda.de; 7 Center for Applied Health Science, Leuphana University Lueneburg, 21335 Lueneburg, Germany; 8 Department of Education, National Taichung University of Education, Taichung 403-06, Taiwan

**Keywords:** COVID-19, coronavirus-related health literacy, Oxford COVID-19 vaccine hesitancy, gender, well-being, depression, school principal, employer, Taiwan

## Abstract

Purposes: To explore the associated factors of COVID-19 vaccine hesitancy and examine psychometric properties of the coronavirus-related health literacy questionnaire (HLS-COVID-Q22) and Oxford COVID-19 Vaccine Hesitancy questionnaire. Methods: An online survey was conducted from 23 June to 16 July 2021 on 387 school principals across Taiwan. Data collection included socio-demographic characteristics, information related to work, physical and mental health, COVID-19 related perceptions, sense of coherence, coronavirus-related health literacy, and vaccine hesitancy. Principal component analysis, correlation analysis, linear regression models were used for validating HLS-COVID-Q22, Oxford COVID-19 Vaccine Hesitancy, and examining the associations. Results: HLS-COVID-Q22 and Oxford COVID-19 Vaccine Hesitancy were found with satisfactory construct validity (items loaded on one component with factor loading values range 0.57 to 0.81, and 0.51 to 0.78), satisfactory convergent validity (item-scale correlations range 0.60 to 0.79, and 0.65 to 0.74), high internal consistency (Cronbach’s alpha = 0.96 and 0.90), and without floor or ceiling effects (percentages of possibly lowest score and highest score <15%), respectively. Low scores of vaccine hesitancy were found in male principals (regression coefficient, B, −0.69; 95% confidence interval, 95%CI, −1.29, −0.10; *p* = 0.023), principals with better well-being (B, −0.25; 95%CI, −0.47, −0.03; *p* = 0.029), and higher HLS-COVID-Q22 (B, −1.22; 95%CI, −1.89, −0.54; *p* < 0.001). Conclusions: HLS-COVID-Q22 and Oxford COVID-19 Vaccine Hesitancy were valid and reliable tools. Male principals and those with better well-being, and higher health literacy had a lower level of vaccine hesitancy. Improving principals’ health literacy and well-being is suggested to be a strategic approach to increase vaccine acceptance for themselves, their staff, and students.

## 1. Introduction

Vaccines against the severe acute respiratory syndrome coronavirus 2 (SARS-CoV-2) infection have been successfully developed with no significantly serious side effects identified [1,2,3,4]. Several new vaccines have been successfully developed and more are to be developed [4,5]. However, hesitancy for COVID-19 vaccines was higher than for other vaccines [6,7], and the vaccine hesitancy in the public has been a great concern to fight COVID-19 [8,9,10,11]. Therefore, improving public acceptance and confidence to get vaccinated is critically important [12,13,14].

A global survey has shown that 61.4% of respondents would accept their employers’ recommendation to take a COVID-19 vaccine [14]. Women were more likely vaccinated as recommended by their employers in Brazil and the United States, while men were more likely to follow their employer’s suggestion in India and South Korea [15]. In addition, in a study involving 1007 Austrians, lack of trust in the government was found to be linked to hesitancy toward a COVID-19 vaccine [16]. Therefore, understanding the leaders’ perception toward the COVID-19 vaccine and associated factors could help to increase the vaccination willingness and uptake of employees and communities.

The hesitancy to get vaccinated against SARS-CoV-2 was associated with health literacy in the general population [17]. Health literacy also plays an important role in controlling the pandemic and infodemic [18]. However, it was underestimated amidst the COVID-19 pandemic [19]. The valid coronavirus-related health literacy questionnaire (HLS-COVID-Q22) was developed to evaluate people’s ability to access, understand, appraise, and apply health-related information in the context of the COVID-19 pandemic [20]. The Oxford COVID-19 Vaccine Hesitancy questionnaire was developed to assess the willingness to take an approved COVID-19 vaccine [13]. The sum score of 7 items was used for analysis and interpretation [13], instead of using only one question and categorical values in other studies [10,21,22,23,24].

Hesitancy for COVID-19 vaccines was investigated in general populations [10], health care workers [25,26,27,28,29,30], students [31,32,33,34,35], and school teachers [36,37]. However, there is no study investigating vaccine hesitancy in school principals who are at the frontline of all school matters and may have an influence on their staff and student’s perceptions and behaviors. Therefore, we conducted an online survey of school leaders to explore factors associated with COVID-19 vaccine hesitancy, and to examine psychometric properties of the HLS-COVID-Q22 questionnaire and the Oxford COVID-19 Vaccine Hesitancy questionnaire.

## 2. Materials and Methods

### 2.1. Study Design and Data Collection

An online survey was conducted on school principals across Taiwan from 23 June to 16 July 2021, as a part of an international COVID-HL research network on school principals [38]. The Ministry of Education and local government agents were contacted to post the online survey web-link on the Principals’ Line group (for internal communications among principals). In addition, the National Association of Primary and Secondary Principals was contacted to post the online survey web-link in the member Line group (for internal communications among the head of City or County Association of Primary and Secondary Principals). The heads then announced the online survey web-link in their Principals’ Line group. We also used Messengers, Facebook, and Email to invite school principals at all levels in our network to fill the online survey. It took about 20–30 min to complete the survey. The data were coded and analyzed anonymously.

### 2.2. Sampling and Sample Size

A network sampling technique was used to recruit the study participants. The calculated sample size was 341 as estimated using the G Power software version 3.1.9.7 for Windows [39], with type I error of 0.05, effect size of 0.1, power of 0.95, and 23 potential predictors in multiple linear regression. In this study, we recruited 413 principals from 3909 schools [40], including 260 from primary school (out of 2631), 84 from junior high school (out of 737), 60 from senior high school and vocational school (out of 513), 9 from school for special children (out of 28). The final sample of 387 principals was used for analysis after excluding 26 outliers. The distribution of the study sample and the total school principals by different locations (north, center, south, east, and outlying islands) and school types (primary school, junior high school, senior high school and vocational school, and school for special children) is presented in Supplementary Material, Table S1. The study sample can be considered representative of the school principal population in Taiwan.

### 2.3. Instruments and Measurements

The Chinese version of questionnaires was used in this survey including sociodemographic factors, work-related factors, physical and mental health-related factors, COVID-19 related perceptions, sense of coherence, coronavirus-related health literacy, and Oxford COVID-19 vaccine hesitancy.

#### 2.3.1. Sociodemographic Factors

We assessed the basic information, including age (year), gender (female vs. male), school types (primary school, junior high school, senior high school or vocational school, school for special children), school locations (north, center, south, east, outlying islands), school size (≤12 classes, 13–24 classes, 25–48 classes, ≥49 classes), according to the regulations of Ministry of Education in Taiwan [41].

#### 2.3.2. Work-Related Factors

School principals were asked whether they were still involved in teaching or not, weekly working hours, and the changes in working hours as compared to that before the COVID-19 pandemic.

#### 2.3.3. Physical and Mental Health-Related Factors

The school principals were asked about their general health status on a 5-point Likert scale from “very bad” to “very good” and dichotomized into “Very bad or bad or moderate” vs. “Good or very good” for analysis. Next, they were asked about the chronic health conditions (no vs. yes), and physical limitations due to chronic conditions (not limited vs. limited).

The symptoms that were similar to COVID-19, or suspected COVID-19 symptoms (S-COVID-19-S) were assessed [42], including fever, cough, and dyspnea, myalgia, fatigue, sputum production, confusion, headache, sore throat, rhinorrhea, chest pain, hemoptysis, diarrhea, and nausea/vomiting. The participants were divided into two groups (with and without S-COVID-19-S), based on their responses.

The level of fear was assessed using the fear of COVID-19 scale (FCoV-19S) [43] which was validated and used in Taiwan [44,45,46]. School principals responded to 7 items on a 5-point Likert scale from 1 = strongly disagree to 5 = strongly agree. The sum scores range from 7 to 35, with a higher score indicating a greater fear. The Cronbach’s alpha value in the current study was 0.90.

Stress was assessed using the 10-item perceived stress scale (PSS-10) that was widely used in international studies [47,48,49]. The tool was also validated and used in the Taiwanese context [50,51], and in the hospital staff during the COVID-19 pandemic [52]. School principals responded to each item on a 5-point Likert scale from 0 = never to 4 = very often. The score of items 4, 5, 7, 8 were reversed. The total scores range from 0 to 40, with a higher score representing higher perceived stress. The Cronbach’s alpha value for the overall scale in the current study was 0.80.

Subjective well-being was assessed using the WHO-5 well-being index with 5 items [53], which has been widely used across 35 countries [54]. The tool was validated and used in the Taiwanese context [55,56,57]. Respondents answered the questions on the 6-point Likert scale from 0 = at no time to 5 = all of the time. The final score (range 0–100) was calculated using the total raw score (range 0–25) multiplied by 4, with 0 reflecting the worst well-being and 100 reflecting the best well-being. The Cronbach’s alpha value in the current study was 0.92.

#### 2.3.4. COVID-19 Related Perceptions

The school principals were asked about the levels of informing on COVID-19 related information “*How well informed do you feel about the COVID-19 related information?*” with 5-point responses ranging from “insufficiently informed” to “very well informed”, and levels of confusion due to COVID-19 related information “*How much confusion do you feel about COVID-19 related information?*” with 4-point responses ranging from “not at all confused” to “very confused” [20]. The response options were dichotomized into “Insufficient/poor/fine informed” vs. “Well or very well informed”, and “Not at all or little confused” vs. “Quite or very confused”, for analysis, respectively.

The perceived COVID-19 threat was assessed using two questions, including “*How concerned are you that you or a family member could get infected with coronavirus in the next 1 year?*” with 4-point responses ranging from “very concerned” to “not concerned at all”, and “*How likely is it that you or a family member could get infected with coronavirus in the next 1 year?*” with 4-point responses ranging from “very likely” to “definitely not” [23]. The response options were dichotomized into “Slightly concerned/not concerned at all” vs. “Very concerned/concerned”, and “Not likely/definitely not” vs. “Very likely/somewhat likely” for analysis, respectively.

#### 2.3.5. Sense of Coherence

Sense of coherence (SOC) was assessed using the 9-item scale that was developed and validated in a previous study [58]. The original scale focuses on the work context (*How do you personally find your current job and work situation in general?*) [58]. This tool was adapted to assess the general living situation amidst the COVID-19 pandemic in the Chinese context [59]. Item response options ranged from 0 to 6. The responses of items 1,3,6,7,9 were reversed for calculating the overall score. The overall score was the average score of 9 items, with higher values indicating a higher SOC [58,59]. The Cronbach’s alpha value in the current study was 0.93.

#### 2.3.6. Coronavirus-Related Health Literacy

Coronavirus-related health literacy was measured using the HLS-COVID-Q22, which includes 22 items and was developed and validated in a previous study [20]. The tool was used to assess the participants’ ability (difficulty or ease) to access, understand, appraise, and apply health-related information in the context of the COVID-19 pandemic [20]. Respondents answered the questions on a 4-point scale ranging from 1 = very difficult to 2 = difficult, 3 = easy, and 4 = very easy. The average scores ranged from 1 to 4 with a higher score presenting a better coronavirus-related health literacy [20].

#### 2.3.7. COVID-19 Vaccine Hesitancy

The COVID-19 vaccine hesitancy was assessed using the Oxford COVID-19 vaccine hesitancy scale with seven items that was validated in a previous study [13]. Each item with specific response options was used and coded from 1 to 5 [60]. A “don’t know” option was excluded from scoring [13]. The sum scores ranged from 7 to 35 with higher scores with higher values indicating a higher level of vaccine hesitancy [12,13].

### 2.4. Ethical Consideration

This study was reviewed and approved by the Research Ethics Committee of National Chengchi University (IRB No. NCCU-REC-202106-I066). All participants were informed about the purposes and importance of the study and voluntarily took the survey.

### 2.5. Statistical Analysis

Since the COVID-19 vaccine hesitancy was an outcome, its normal distribution was checked. The histogram, normal Q-Q plot, and box plot showed that the COVID-19 vaccine hesitancy scores were approximately normally distributed, with a skewness of 0.179 (SE = 0.124) [61]. After excluded 26 outliners, the final sample for analysis was 387 school principals. The one-way ANOVA tests were performed to explore the distribution of COVID-19 vaccine hesitancy by different categories of independent variables.

#### 2.5.1. Psychometric Properties of the HLS-COVID-Q22 and COVID-19 Vaccine Hesitancy

The construct validity was examined using the principal component analysis (PCA) with the oblique rotation (Promax) to evaluate the construct of the HLS-COVID-Q22 and COVID-19 Vaccine Hesitancy. The Kaiser-Meyer Olkin criterion and Bartlett’s Test of Sphericity value were used to determine the sampling adequacy, and the suitability of the data for PCA, respectively [62].

The correlations between scale and its items were checked using Spearman’s correlation to assess the item-scale convergent validity [63,64].

Floor and ceiling effects were calculated using the percentages of participants with the possibly lowest score and highest score [65].

The internal consistency of the HLS-COVID-Q22 and COVID-19 Vaccine Hesitancy were estimated using Cronbach’s alpha test. A Cronbach’s alpha value of ≥0.70 was designated for satisfactory reliability [66].

#### 2.5.2. Associated Factors of COVID-19 Vaccine Hesitancy

Associated factors of COVID-19 vaccine hesitancy were investigated using bivariate and multivariate linear regression models. To minimize residual effects of confounders, all the factors in the bivariate model were analyzed in the multivariate model [67]. The variance inflation factor (VIF) values were checked to detect multicollinearity. Data were analyzed using the IBM SPSS Version 20.0 for Windows (IBM Corp., Armonk, NY, USA). A *p*-value < 0.05 was set as the significance level.

## 3. Results

### 3.1. Study Participants’ Characteristics

A sample of 387 school principals was analyzed, the mean age was 52.9 ± 4.8 years (ranged from 37 to 70), 64.9% were men, 27.4% were involved in teaching. Average working hours a week were 48.9 ± 14.9, 77.2% worked more than 40 h a week, 17.8% worked more than before the pandemic. The means of coronavirus-related health literacy and COVID-19 vaccine hesitancy were 3.2 ± 0.4 and 11.2 ± 2.7, respectively (Table 1).

### 3.2. Psychometric Properties of HLS-COVID-Q22 and COVID-19 Vaccine Hesitancy

The KMO values of HLS-COVID-Q22 and COVID-19 Vaccine Hesitancy were 0.95, and 0.85 indicating sample adequacy (≥0.6) [62]. Bartlett’s Test of Sphericity values of HLS-COVID-Q22 and COVID-19 Vaccine Hesitancy were <0.001, which determined the suitability of the data for PCA [62]. Twenty-two items of the HLS-COVID-Q22, and 7 items of COVID-19 Vaccine Hesitancy were strongly loaded on one component each and explained 52.3%, and 49.4% of the scale variance, respectively. The HLS-COVID-Q22 items’ and COVID-19 Vaccine Hesitancy items’ factor loading values ranged from 0.57 to 0.81 (Table 2), and 0.51 to 0.78 (Table 3), respectively. This indicates a satisfactory construct validity [62].

The correlations between HLS-COVID-Q22 and COVID-19 Vaccine Hesitancy and their items ranged from 0.60 to 0.79 (Table 2), and 0.65 to 0.74 (Table 3), respectively, indicating satisfactory convergent validity [63,64]. In addition, the Cronbach’s alpha values for HLS-COVID-Q22 and COVID-19 Vaccine Hesitancy were 0.96 and 0.90 respectively, reflecting a high level of internal consistency. There were no significant floor and ceiling effects with proportions of the lowest potential response, and the highest potential response of less than 15% for HLS-COVID-Q22 (Table 2), and COVID-19 Vaccine Hesitancy (Table 3) [65].

### 3.3. Associated Factors of COVID-19 Vaccine Hesitancy

In the multivariate analysis, male school principals had lower scores of vaccine hesitancy (regression coefficient, B, −0.69; 95% confidence interval, 95%CI, −1.29, −0.10; *p* = 0.023) as compared to female respondents. Principals of junior high schools had higher scores of vaccine hesitancy (B, 0.75; 95%CI, 0.02, 1.47; *p* = 0.043) as compared to those from primary schools. Respondents with higher scores of subjective well-being (a 10-score increment) had lower scores of vaccine hesitancy (B, −0.25; 95%CI, −0.47, −0.03; *p* = 0.029). Finally, school principals with higher HL scores had lower vaccine hesitancy scores (B, −1.22; 95%CI, −1.89, −0.54; *p* < 0.001; Table 4). The adjusted R^2^ = 0.138, the variance inflation factor (VIF) ranged 1.12 to 3.05, indicating no multicollinearity [62].

## 4. Discussion

The HLS-COVID-Q22 and COVID-19 Vaccine Hesitancy questionnaires were valid and reliable tools for assessing coronavirus-related health literacy and vaccine hesitancy in Taiwan during the pandemic. Both tools showed satisfactory construct validity [62], convergent validity [63,64], and reliability [68], and no floor or ceiling effect [65].

A previous study showed that vaccine willingness was low in Taiwan due to the relatively safe status of COVID-19 infection [28]. However, in our study, the prevalence of vaccine acceptance was relatively high (95.6% “probably” and “definitely” take a COVID-19 vaccine if offered, Supplementary Material, Table S2). In addition, the overall COVID-19 vaccine hesitancy score in our study (11.2 ± 2.7) was lower than that in a previous study (13.6 ± 7.3) that used the same tool [13]. The difference might be accounted for by survey time, as our survey was conducted during the peak of the pandemic in Taiwan [69]. This also reflects a fact that the school principals (a very highly educated population with high socioeconomic status, as compared to the general population) might have a higher willingness to get a jab against COVID-19. In addition, a previous finding showing that schoolteachers were likely or very likely to accept a COVID-19 vaccine [36].

In the current study, male principals had lower vaccine hesitancy scores than female respondents. This is consistent with previous studies’ findings that vaccine hesitancy is higher in women [22,36,70]. This could be explained that women had more concerns about safety [71,72], and reproductive health [73] during the pandemic. Inconsistently, in some countries, women were more likely to accept a vaccine (e.g., France, Germany, Sweden, and Russia) as they are gatekeepers for their family’s health-related decisions, and with higher empathy levels for their family safety [15]. In addition, the principals of junior high schools had higher scores of vaccine hesitancy than those of primary schools in the current study. The finding provides evidence for the COVID-19 vaccine promotion strategies suggesting more attention to female and junior high school principals. The COVID-19 vaccine hesitancy in school principals could be influenced by socio-demographic and health beliefs as found in a previous study conducted on the schoolteachers [37]. Social and organizational factors were also found to have certain influences on the public’s attitude toward vaccines [21].

In our study, better general well-being was associated with lower COVID-19 vaccine hesitancy. A previous study showed that healthcare workers with depression had a lower likelihood of taking a COVID-19 vaccine [25]. In turn, when they took the vaccine, the vaccine hesitancy showed a negative impact on their mental health [74]. Fear of COVID-19 and perceived stress were not found to be associated with vaccine hesitancy in our study. In contrast, a previous study showed that fear and anxiety were associated with higher COVID-19 vaccine acceptance [75]. The perceived COVID-19 threat was not associated with vaccine hesitancy in the current study. However, a previous study showed that people who perceived a higher COVID-19 risk had a higher likelihood of willingness to vaccinate [28]. In addition, people with greater health concerns about COVID-19 had a lower hesitant level [70].

In the present study, higher health literacy was strongly associated with lower COVID-19 vaccine hesitancy. In previous studies, the willingness to get a COVID-19 vaccine was determined by knowledge about coronavirus transmission [76], and COVID-19 [77]. The ability to detect fake news and higher health literacy was associated with a higher likelihood of a COVID-19 vaccine acceptance [17]. Conspiracy beliefs and myths about the COVID-19 vaccine affect COVID-19 vaccination [78]. Therefore, to increase the willingness to get vaccinated against COVID-19, it is suggested to improve individuals’ health literacy, and ability to detect fake news [17], by addressing the source of information [79], continuously providing reliable information, improving communication to address the root causes of mistrust, using a diverse range of policies and technologies [80], optimizing the official communication in the context of vaccine misinformation [81]. It is also important to implement health literacy intervention on the individual, interpersonal, and organizational levels [82], ideally facilitated by sustainable policy efforts both locally and nationally.

The level of being informed or confused about COVID-19 information was not significantly associated with vaccine hesitancy in our study. This was similar to previous evidence such that transparently informing people of the vaccination limitations did not affect vaccination intentions [83]. In addition, the characteristics of different vaccines potentially influenced the attitudes of the public towards vaccine acceptance [21]. People hesitated to receive a COVID-19 vaccine because of other reasons, e.g., fears of injection [84], or concerns about vaccine efficacy and fear of side effects [78]. Therefore, to increase the vaccination intentions, a simple message mentioning the vaccine efficacy is possibly influenced by context that might be helpful [85]. A simple “nudge” message could show the power to increase COVID-19 vaccine uptake [86,87].

Our study has some limitations. Firstly, this was a cross-sectional study in which the causal relationship cannot be generated. Secondly, the study was conducted during the peak of the pandemic in Taiwan [69], the measurement of COVID-19 vaccine hesitancy may be biased. However, we collected data from different regions with different infection rates, and vaccine hesitancy was not significantly differed by school locations. Finally, the sample investigated was about 9.9% (387/3909) of total school principals that may affect the generalizability of findings. However, the study can raise the phenomena and provide immediate evidence for appropriate interventions.

## 5. Conclusions

The HLS-COVID-Q22 and Oxford COVID-19 Vaccine Hesitancy are valid and reliable instruments to measure coronavirus-related health literacy and vaccine hesitancy, respectively, among educational leaders. We found that male principals, those with better well-being, and higher health literacy had a lower level of vaccine hesitancy. Strategical interventions are suggested to improve the perception of school principals toward COVID-19 vaccine. These further influences behaviors of school teachers and students regarding vaccination which helps to contain the pandemic.

## Figures and Tables

**Table 1:** School principals’ characteristics, their works, health, COVID-19 related information and health literacy, sense of coherence, and vaccine hesitancy.

Variables	Total (*n* = 387)	COVID-19 Vaccine Hesitancy	
	*n* (%)	Mean ± SD	*p*-Value *
Age, mean ± SD, 37–70 years	52.9 ± 4.8		
Gender			0.011
Women	136 (35.1)	11.7 ± 2.8	
Men	251 (64.9)	11.0 ± 2.6	
School type			0.020
Primary school	243 (62.8)	11.1 ± 2.7	
Junior high school	77 (19.9)	12.0 ± 2.7	
Senior high school and vocational school	58 (15.0)	10.7 ± 2.5	
School for special children	9 (2.3)	12.1 ± 2.5	
School location			0.359
North	117 (30.2)	11.3 ± 2.7	
Center	127 (32.8)	11.0 ± 2.6	
South	80 (20.7)	11.5 ± 2.8	
East	38 (9.8)	11.6 ± 2.5	
Outlying islands	25 (6.5)	10.6 ± 2.4	
School Size			0.221
≤12 classes	142 (36.7)	11.5 ± 2.8	
13–24 classes	71 (18.3)	11.3 ± 2.7	
25–48 classes	98 (25.3)	10.8 ± 2.7	
≥49 classes	76 (19.7)	11.2 ± 2.4	
Involving teaching			0.341
No	281 (72.6)	11.3 ± 2.7	
Yes	106 (27.4)	11.0 ± 2.7	
Weekly working hours, mean ± SD	48.9 ± 14.9		0.857
<40 h	32 (8.3)	11.0 ± 3.1	
40 h	56 (14.5)	11.3 ± 2.6	
>40 h	299 (77.2)	11.2 ± 2.6	
Weekly working hours changes			0.745
Less than before the pandemic	113 (29.2)	11.4 ± 2.7	
About the same	205 (53.0)	11.1 ± 2.6	
More than before the pandemic	69 (17.8)	11.2 ± 2.8	
General health status			0.024
Very bad or bad or moderate	155 (40.1)	11.6 ± 2.6	
Good or very good	232 (69.9)	11.0 ± 2.7	
Chronic health conditions			0.583
No	237 (61.2)	11.2 ± 2.8	
Yes	150 (38.8)	11.3 ± 2.7	
Physical limitation due to chronic conditions			0.725
Not limited	279 (72.1)	11.2 ± 2.6	
Limited	108 (27.9)	12.3 ± 2.7	
S-COVID-19-S			0.552
No	246 (63.6)	11.2 ± 2.7	
Yes	141 (36.4)	11.3 ± 2.5	
FCoV-19S	17.5 ± 5.3		
Perceived stress, mean ± SD	12.7 ± 4.5		
WHO-5 Wellbeing Index, mean ± SD	69.5 ± 15.4		
Level of informing on COVID-19 related information			0.246
Insufficient/poor/fine informed	44 (11.4)	11.7 ± 3.3	
Well or very well informed	343 (88.6)	11.2 ± 2.6	
Level of confusion due to COVID-19 related information			0.187
Not at all or little confused	351 (90.7)	11.2 ± 2.6	
Quite or very confused	36 (9.3)	11.8 ± 3.3	
Level of concern about getting infected			0.893
Slightly concerned/not concerned at all	129 (33.3)	11.2 ± 2.6	
Very concerned/concerned	258 (66.7)	11.2 ± 2.7	
Perceived likelihood of getting infected			0.849
Not likely/definitely not	159 (41.1)	11.2 ± 2.7	
Very likely/somewhat likely	228 (58.9)	11.2 ± 2.7	
Sense of coherence, mean ± SD	4.2 ± 1.1		
Coronavirus-related HL, mean ± SD	3.2 ± 0.4		
Vaccine Hesitancy, mean ± SD	11.2 ± 2.7		

Abbreviations: COVID-19, coronavirus disease 2019; SD, standard deviation; S-COVID-19-S, suspected COVID-19 symptoms; FCoV-19S, fear of COVID-19; WHO, world health organization; HL, health literacy. * Results of one-way ANOVA test.

**Table 2:** Construct, convergent, internal consistency, floor and ceiling effects of Coronavirus-related health literacy (*n* = 387).

	HLS-COVID-Q22
Factor loading values *	Component 1
1. find information about the coronavirus on the internet?	0.57
2. find information on the internet about protective behaviors that can help to prevent infection with the coronavirus?	0.66
3. find information in newspapers, magazines and on TV about behaviors that can help to prevent infection with the corona-virus?	0.64
4. find out information how to recognize if I am likely to be infected with the coronavirus?	0.72
5. find information on how to find professional help in case of coronavirus infection?	0.78
6. find information on how I much I am at risk for infection with coronavirus?	0.75
7. understand your doctor’s, pharmacist’s or nurse’s instructions on protective measures against coronavirus infection?	0.71
8. understand recommendations of authorities regarding protective measures against coronavirus infection?	0.67
9. understand advice from family members or friends regarding protective measures against coronavirus infection?	0.76
10. understand information in the media on how to protect myself against coronavirus infection?	0.72
11. understand risks of the coronavirus that I find on the internet?	0.77
12. understand risks of the coronavirus that I find in newspapers, magazines or on TV?	0.77
13. judge if information on coronavirus and the coronavirus epidemic in the media is reliable?	0.73
14. judge which behaviors are associated with higher risk of coronavirus infection?	0.77
15. judge what protective measures you can apply to prevent a coronavirus infection?	0.81
16. judge how much I am at risk for a coronavirus infection?	0.72
17. judge if I have been infected with coronavirus?	0.60
18. decide how you can protect yourself from coronavirus infection based on information in the media?	0.76
19. follow instructions from your doctor or pharmacist regarding how to handle the coronavirus situation?	0.73
20. use information the doctor gives you to decide how to handle an infection with coronavirus?	0.73
21. use media information to decide how to handle an infection with coronavirus?	0.77
22. to behave in a way to avoid infecting others?	0.73
Percentage of variance, %	52.3%
Item-scale convergent validity, mean of Rho (range)	0.72 (0.60–0.79)
Internal consistency, Cronbach’s alpha	0.96
Floor effects, %	0.00
Ceiling effect, %	5.90

Abbreviations: HLS-COVID-Q22, coronavirus-related health literacy; Rho, Spearman’s correlation coefficient. * Principal component analysis using Promax rotation method.

**Table 3:** Construct, convergent, internal consistency, floor and ceiling effects of COVID-19 vaccine hesitancy (*n* = 387).

	COVID-19 Vaccine Hesitancy
Factor loading values *	Component 1
1. Would you take a COVID-19 vaccine if offered? …	0.51
2. If there is a COVID-19 vaccine available,…	0.73
3. I would describe my attitude towards receiving a COVID-19 vaccine as:…	0.76
4. If a COVID-19 vaccine was available at my local pharmacy, I would:…	0.72
5. If my family or friends were thinking of getting a COVID-19 vaccination, I would:…	0.72
6. I would describe myself as:…	0.78
7. Taking a COVID-19 vaccination is:…	0.66
Percentage of variance, %	49.4%
Item-scale convergent validity, mean of Rho (range)	0.70 (0.65–0.74)
Internal consistency, Cronbach’s alpha	0.90
Floor effects, %	11.60
Ceiling effect, %	0.00

Abbreviations: COVID-19, coronavirus disease 2019; Rho, Spearman’s correlation coefficient. * Principal component analysis using Promax rotation method.

**Table 4:** Associated factors of COVID-19 vaccine hesitancy via bivariate and multivariate linear regression analyses (*n* = 387).

Variables	COVID-19 Vaccine Hesitancy			
	Bivariate		Multivariate	
	B (95% CI)	*p*	B (95% CI)	*p*
Age	0.01 (−0.05, 0.06)	0.789	0.01 (−0.05, 0.07)	0.697
Gender				
Women	Reference		Reference	
Men	−0.72 (−1.27, −0.16)	0.011	−0.69 (−1.29, −0.10)	0.023
School type				
Primary school	Reference		Reference	
Junior high school	0.89 (0.21, 1.57)	0.010	0.75 (0.02, 1.47)	0.043
Senior high school and vocational school	−0.35 (−1.10, 0.41)	0.371	−0.14 (−0.99, 0.71)	0.744
School for special children	1.04 (−0.72, 2.8)	0.246	0.84 (−0.97, 2.65)	0.362
School location				
North	Reference		Reference	
Center	−0.33 (−1.00, 0.34)	0.332	−0.43 (−1.11, 0.26)	0.226
South	0.20 (−0.56, 0.96)	0.596	0.16 (−0.65, 0.96)	0.700
East	0.27 (−0.71, 1.25)	0.586	0.13 (−0.92, 1.17)	0.810
Outlying islands	−0.75 (−1.90, 0.41)	0.204	−0.68 (−1.88, 0.52)	0.265
School Size				
≤12 classes	Reference		Reference	
13–24 classes	−0.15 (−0.91, 0.61)	0.702	−0.20 (−0.99, 0.59)	0.622
25–48 classes	−0.72 (−1.41, −0.03)	0.040	−0.67 (−1.44, 0.09)	0.085
≥49 classes	−0.29 (−1.03, 0.45)	0.446	−0.19 (−1.06, 0.68)	0.671
Involving teaching				
No	Reference		Reference	
Yes	−0.29 (−0.89, 0.31)	0.341	−0.12 (−0.73, 0.5)	0.710
Weekly working hours				
<40 h	−0.28 (−1.45, 0.88)	0.635	−0.35 (−1.53, 0.84)	0.563
40 h	Reference		Reference	
>40 h	−0.01 (−0.77, 0.76)	0.981	−0.05 (−0.84, 0.75)	0.906
Weekly working hours changes				
Less than before the pandemic	0.24 (−0.38, 0.86)	0.444	0.31 (−0.31, 0.94)	0.324
About the same	Reference		Reference	
More than before the pandemic	0.10 (−0.63, 0.83)	0.788	0.13 (−0.61, 0.88)	0.726
Self-endangering work behavior	0.18 (−0.31, 0.67)	0.473	0.03 (−0.55, 0.61)	0.913
General health status				
Very bad or bad or moderate	Reference		Reference	
Good or very good	−0.62 (−1.16, −0.08)	0.024	−0.27 (−0.94, 0.40)	0.436
Chronic health conditions				
No	Reference		Reference	
Yes	0.15 (−0.39, 0.70)	0.583	0.22 (−0.46, 0.90)	0.520
Physical limitation due to chronic conditions				
Not limited	Reference		Reference	
Limited	0.11 (−0.49, 0.70)	0.725	−0.44 (−1.22, 0.34)	0.268
S-COVID-19-S				
No	Reference		Reference	
Yes	0.17 (−0.39, 0.72)	0.552	0.05 (−0.54, 0.64)	0.864
FCoV-19S	−0.02 (−0.07, 0.03)	0.360	−0.06 (−0.12, 0.00)	0.059
Perceived stress	0.01 (−0.05, 0.07)	0.654	−0.06 (−0.13, 0.02)	0.152
WHO-5 Wellbeing Index, a 10-score increment	−0.28 (−0.45, −0.11)	0.001	−0.25 (−0.47, −0.03)	0.029
Level of informing on COVID-19 related information				
Insufficient/poor/fine informed	Reference		Reference	
Well or very well informed	−0.50 (−1.33, 0.34)	0.246	−0.04 (−0.94, 0.87)	0.936
Level of confusion due to COVID-19 related information				
Not at all or little confused	Reference		Reference	
Quite or very confused	0.62 (−0.30, 1.53)	0.187	0.44 (−0.56, 1.44)	0.386
Level of concern about getting infected				
Slightly concerned/not concerned at all	Reference		Reference	
Very concerned/concerned	0.04 (−0.53, 0.60)	0.893	−0.02 (−0.98, 0.93)	0.960
Perceived likelihood of getting infected				
Not likely/definitely not	Reference		Reference	
Very likely/somewhat likely	0.05 (−0.49, 0.59)	0.849	0.01 (−0.90, 0.93)	0.977
Sense of coherence	−0.15 (−0.40, 0.10)	0.241	0.00 (−0.28, 0.27)	0.986
Coronavirus-related HL	−1.27 (−1.87, −0.67)	<0.001	−1.22 (−1.89, −0.54)	<0.001

Abbreviations: COVID-19, coronavirus disease 2019; B, regression coefficient; CI, confidence interval; S-COVID-19-S, suspected COVID-19 symptoms; FCoV-19S, fear of COVID-19; WHO, world health organization; HL, health literacy.

## Data Availability

Data will be available on the reasonable request from the corresponding author.

## Supplementary Materials

The following are available online at https://www.mdpi.com/article/10.3390/vaccines9090985/s1, Table S1: The distribution of study sample and total school principals by school locations and types (*n* = 387), Table S2: The distribution of school principals by different COVID-19 vaccine hesitancy items (*n* = 387).
