# Supplementary material for: Oxford COVID-19 Vaccine Hesitancy in School Principals: Impacts of Gender, Well-Being, and Coronavirus-Related Health Literacy
Source: Vaccines (Basel). 2021 Sep 3;9(9):985. doi: 10.3390/vaccines9090985 (PMC8471420; doi:10.3390/vaccines9090985)
Supplement: Supplementary file 1 [file vaccines-09-00985-s001.zip › vaccines-1351452-supplementary.pdf]

Table S1. The distribution of study sample and total school principals by school locations and types (n = 387).

Study sample/total school principal ratio		School location						
		**North**	**Center**	**South**	**East**	**Outlying islands**	**Subtotal**	**%**
**School type**	**Primary school**	71/794	74/820	53/763	29/191	16/63	243/2631	9.2
	**Junior high school**	16/258	34/203	17/208	5/44	5/24	77/737	10.4
	**Senior high school and vocational school**	25/212	16/132	9/141	4/23	4/5	58/513	11.3
	**School for special children**	5/9	3/9	1/8	0/2	0/0	9/28	32.1
	**Subtotal**	117/1273	127/1164	80/1120	38/260	25/92	387/3909	9.9
	%	9.2	10.9	7.1	14.6	27.2	9.9	

Table S2. The distribution of school principals by different COVID-19 vaccine hesitancy items (n = 387).

Items	Responses	n	%
1. Would you take a COVID-19 vaccine if offered?	□1. Definitely	259	66.9
	□2. Probably	111	28.7
	□3. I may or I may not	14	3.6
	□4. Probably not	1	0.3
	□5. Definitely not	2	0.5
	□6. Don't know	0	0.0
2. If there is a COVID-19 vaccine available	□1. I will want to get it as soon as possible	145	37.5
	□2. I will take it when offered	233	60.2
	□3. I'm not sure what I will do	6	1.6
	□4. I will put off (delay) getting it	3	0.8
	□5. I will refuse to get it	0	0.0
	□6. Don't know	0	0.0
3. I would describe my attitude towards receiving a COVID-19 vaccine as:	□1. Very keen	78	20.2
	□2. Pretty positive	266	68.7
	□3. Neutral	42	10.9
	□4. Quite uneasy	0	0.0
	□5. Against it	0	0.0
	□6. Don't know	1	0.3
4. If a COVID-19 vaccine was available at my local pharmacy, I would:	□1. Get it as soon as possible	262	67.7
	□2. Get it when I have time	116	30.0
	□3. Delay getting it	8	2.1
	□4. Avoid getting it for as long as possible	0	0.0
	□5. Never get it	0	0.0
	□6. Don't know	1	0.3
5. If my family or friends were thinking of getting a COVID-19 vaccination, I would:	□1. Strongly encourage them	131	33.9
	□2. Encourage them	223	57.6
	□3. Not say anything to them about it	28	7.2
	□4. Ask them to delay getting the vaccination	3	0.8
	□5. Suggest that they do not get the vaccination	0	0.0
	□6. Don't know	2	0.5
6. I would describe myself as:	□1. Eager to get a COVID-19 vaccine	94	24.3
	□2. Willing to get the COVID-19 vaccine	283	73.1
	□3. Not bothered about getting the COVID-19 vaccine	9	2.3
	□4. Unwilling to get the COVID-19 vaccine	0	0.0
	□5. Anti-vaccination for COVID-19	0	0.0
	□6. Don't know	1	0.3
7. Taking a COVID-19 vaccination is:	□1. Really important	230	59.4
	□2. Important	148	38.2
	□3. Neither important nor unimportant	8	2.1
	□4. Unimportant	0	0.0
	□5. Really unimportant	0	0.0
	□6. Don't know	1	0.3
